# Sesqui-Centennial Celebration

**Published:** 1880-10

**Authors:** 


SESQUI-CENTENNIAL CELEBRATION. — One hundred and fifty years have been rolled upon the scroll of the unrecallable past since the founding of this great and prosperous city, and the grand and magnificent pageants, cavalcades and street displays, occurring in commemoration thereof, during this centennial week, exceed in gorgeousness and beauty any similar spectacles ever presented before in this country! The whole city is in holiday attire, and all mechanical business operations are suspended, in order that the various trades may participate in the festivities of the occasion. The forms of the present issue were all worked off save the last one, ere the advent of the celebration, but nothing further can be done until its close. We therefore trust our numerous friends and readers will excuse the unexpected delay of the present number. We hope to furnish them with such items of interest, of a medical character, as we may be able to glean during the great celebration, in the next issue of the journal.

THE ARTICLE on Blood-Letting was published in the Philadelphia *Medical and Surgical Reporter*, in January, 1872, and had the effect of calling the attention of the profession to the vast importance of the remedy. Many leading members began soon after to speak and write in advocacy of it. It is re-published in the present issue of this journal at the solicitation of medical friends, who believe that much good will result therefrom.

